# Health Data for Linguistic Minority Group Research in Canada: Proof-of-Concept Centralized Health Care Metadata Repository Development and Usability Study

**DOI:** 10.2196/77242

**Published:** 2026-02-09

**Authors:** Vincent Martin-Schreiber, Cayden Peixoto, Ricardo Batista, Christopher Belanger, Peter Tanuseputro, Amy T Hsu, Lise M Bjerre

**Affiliations:** 1 Faculty of Health Sciences University of Ottawa Ottawa, ON Canada; 2 School of Epidemiology and Public Health Faculty of Medicine University of Ottawa Ottawa, ON Canada; 3 Akausivik Inuit Family Health Team Ottawa, ON Canada; 4 Telfer School of Management University of Ottawa Ottawa, ON Canada; 5 Department of Family Medicine and Primary Care University of Hong Kong Hong Kong China (Hong Kong); 6 Bruyère Health Research Institute Ottawa, ON Canada; 7 Institut du Savoir Montfort Ottawa, ON Canada; 8 Department of Family Medicine Faculty of Medicine University of Ottawa Ottawa, ON Canada

**Keywords:** metadata, metadata repository, variables, language, linguistic

## Abstract

**Background:**

Language barriers between Canadian patients and health care providers are associated with poorer health outcomes, including decreased patient safety and quality of care, misdiagnosis and longer treatment initiation times, and increased mortality. However, research exploring language as a social determinant of health is limited, as Canadian health data are scattered across many jurisdictions, each with its own policies and procedures. This fragmentation makes it difficult for researchers to identify, locate, and use existing data. This paper presents the results of a pilot study that attempts to address this gap by creating a metadata repository (MDR) to act as a central source of information about what data are available at which data holdings across Canada.

**Objective:**

This project aimed to (1) create a proof-of-concept MDR for Canadian health data at the variable level; (2) identify and label language-related variables existing within the MDR data; and (3) develop an interactive, public-facing web application to let users browse and search the MDR.

**Methods:**

Metadata were collected from 5 Canadian health data sources, including 4 provincial data holdings and 1 national survey, and pooled to create a data repository. Then, we performed bottom-up labeling of language-related variables within the pooled metadata by first using a search string algorithm across all variable labels, names, and definitions and then consensus screening these variables using a derived, standardized definition of language or linguistic variables. Using the *Shiny* web framework in R, we then developed an openly accessible web application to allow users to search the proof-of-concept MDR.

**Results:**

A total of 850,343 variables were collected and included in the repository, with most coming from Ontario (n=712,037, 83.7%) and Manitoba (n=97,051, 11.4%) provincial data holdings. Among all variables in the repository, 213,696 (25.1%) were confirmed to be language related.

**Conclusions:**

Developing a national MDR would be a transformative opportunity for Canadian researchers to leverage the full scope of Canadian health administrative data. Although a top-down approach with consistent engagement of and collaboration between provincial data holdings and federal data agencies is ideal to develop a national MDR, this study demonstrates the feasibility of a bottom-up approach in contributing to this overarching goal.

## Introduction

### Background

Canada’s publicly funded health care system generates a vast amount of data covering factors as wide ranging as pharmacy or prescription records, laboratory results, and health care services [1,2]. These data hold immense potential for health care research and for health policy and planning. However, because health care is administered differently across each of Canada’s provinces and territories, the data are scattered across a large number of agencies and institutions, each with its own data policies and procedures [3]. This makes it difficult for researchers to access Canada’s provincial health data and also creates a more fundamental problem—it is often difficult for researchers to even discover what types of data are available, where they are held, and how to access them. This fragmentation has contributed to significant differences in the availability and accessibility of administrative and other health data across provinces, posing a major challenge for interprovincial or pan-Canadian health care research. This “data fragmentation” can create particular problems for health care research related to patient and health care provider language abilities.

Language as a social determinant of health is an important and emerging topic in health research [4,5], and language barriers between Canadian patients and health care providers are associated with misdiagnosis and longer treatment initiation times [6]; negative experiences for patients [7,8] and physicians [9,10]; and, in hospital settings, decreased patient safety and quality of care [11,12] as well as increased mortality [13]. This issue is of specific concern in Canada, an officially bilingual country in which 76.1% of the population are native English speakers, 22% are native French speakers, and 18% are bilingual [14]. Although French speakers and English speakers can be found across the country, most French speakers live in the provinces of Quebec and New Brunswick. Despite the importance of language-related health research, the data fragmentation described previously makes it difficult and time consuming to even discover what language-related data are available, let alone access and analyze them. This paper presents the results of a pilot study that attempts to bridge this gap by creating a “metadata repository” (MDR) to serve as a central source of information about which data are available at which locations across Canada.

Metadata can be defined as “data about data” [15], and for this project, we sought to create a repository of variable-level metadata. In this context, variable-level metadata include information such as the institution holding the variable, the larger collection or “library” to which it belongs, and a plaintext description. To help illustrate the utility of MDR in light of Canada’s bilingual health care context, we put a special focus on identifying language-related variables. In addition to our final metadata dataset, we also created an interactive public-facing web application to let users browse and search the repository.

We discuss the current state of health data and metadata management in Canada and outline the principles and scope guiding our pilot project subsequently.

### Current Initiatives in Canada

There are currently 2 main health metadata initiatives in Canada: the Health Data Research Network (HDRN) Canada’s Data Access Support Hub (DASH) and the Strategy for Patient-Oriented Research (SPOR) Canadian Data Platform (CDP). The HDRN is a pan-Canadian network of health data–holding organizations, and it established DASH [16] to guide researchers and streamline access to data held by its members. However, DASH only helps researchers access data housed at member organizations of HDRN Canada, and its services are not free to use.

The SPOR CDP, announced in 2019 by Canada’s Ministry of Health, is intended to function as a single portal for researchers to request access to administrative, clinical, and social data from sources across the country [17]. To achieve this goal, the SPOR CDP aims to harmonize and validate definitions for key analytic variables (eg, chronic diseases) while expanding the sources, types, and linkages of data available to researchers (eg, social data). Standardizing data definitions allows information exchanged between data holdings to be equally understood by all parties, a concept known as semantic interoperability [18]. Semantic interoperability is especially important as it allows researchers to combine datasets. Canada is known to lag in health data interoperability [19-21], and the development of metadata standards, a set of guidelines that establish a common way of structuring and understanding data [15], would be very helpful. However, the CDP platform was originally announced as a 7-year initiative and is still ongoing as of 2026.

In addition to larger metadata projects, some data-holding organizations also have public-facing websites that allow users to search their metadata. For example, the Institute for Clinical Evaluative Sciences (ICES) in Ontario provides a publicly accessible data dictionary of their metadata that is searchable at the variable level [22]. Although such resources can be helpful, they lead to the problem of data fragmentation described previously, as researchers must visit each organization’s website and consolidate results themselves.

Although there are clear use cases for larger projects such as DASH or the CDP and smaller, institution-level metadata websites, they do not offer a free-to-use and up-to-date repository of health metadata from across Canada. The goal of this study is to take the initial steps toward bridging this gap.

### A National MDR: Pilot Project Principles

In this study, we were guided by 2 sets of principles: the principles of findability, accessibility, interoperability, and reusability (FAIR) data stewardship [23] and a bottom-up principle of researcher-driven development.

The FAIR principles were developed by Wilkinson et al [23] to address the challenges in managing large amounts of data. The FAIR principles stipulate that both data and metadata should be findable, accessible, interoperable, and reusable by researchers [23,24]. Clearly, the fragmented landscape of Canadian administrative health data does not adhere to FAIR principles in this sense, which creates what we view as unnecessary delays and roadblocks to potentially life-saving research.

We also postulate that there is a useful role for researchers to play in creating a pragmatic and useful national MDR within the current Canadian health data landscape. Given the size and complexity of administrative health databases and the dappled policy environment governing data access across Canada, creating an MDR through the top-down approach at the organizational level would take a large degree of coordination, political will, and resources to harmonize data selection, definition, collection, and sharing procedures across all provincial and territorial health data holdings. Although an MDR built through top-down standardization would be ideal, there is no guarantee that one will be available in Canada soon.

According to the Public Health Agency of Canada, federal, provincial, and territorial governments are currently working to improve the sharing of public health information [25]. However, a data-sharing agreement between these governments is not expected until the end of 2026, with bilateral agreements to follow and then a lengthy process of harmonizing definitions and processes across the data holdings. In the meantime, a simpler solution built by and for researchers has the potential to provide value now.

## Methods

### Data Sources and Data Collection

To ensure that our proof-of-concept MDR is robust and inclusive, we aimed to include metadata from a variety of national and provincial administrative health data sources. Administrators and data custodians at national and provincial data holdings (Table 1) were contacted via email between January 2023 and September 2023 to request access to the metadata from all held administrative health datasets, ideally in a raw data format such as CSV. Among the data custodians contacted, metadata were provided by or accessible from the ICES [26], the Manitoba Centre for Health Policy (MCHP) [27], the Institut de la statistique du Québec [28], and the New Brunswick Institute for Research, Data and Training [29]. We also obtained and included metadata from the Canadian Longitudinal Study on Aging [30].

Metadata files were obtained from MCHP and the New Brunswick Institute for Research, Data and Training. For the other 4 data holdings, data scraping [31] was performed by a member of the research team (VM-S) to extract metadata from publicly available online sources and data dictionaries. Detailed explanations of how data were collected from each data holding are provided in Multimedia Appendix 1. Once the metadata from the 5 included data holdings were pooled into a single CSV file, the metadata were organized according to commonly reported data elements across sources, including data holding, dataset name, dates available, variable label, variable name, and variable definition, as reported by the respective data source.

**Table 1:** Data dictionary availability from administrative health data custodians by province.

Province	Data custodian	Publicly accessible data dictionary or catalog
Alberta	Alberta Health	Yes^a^
BC^b^	Population Data BC	Yes
Manitoba	Manitoba Centre for Health Policy	Yes
New Brunswick	New Brunswick Institute for Research, Data and Training	Yes
Newfoundland and Labrador	Newfoundland and Labrador Centre for Health Information	Yes
Nova Scotia	Health Data Nova Scotia	Yes^a^
Ontario	Institute for Clinical Evaluative Sciences	Yes
PEI^c^	Health PEI	No
Quebec	Régie de l’assurance maladie du Québec	No
Saskatchewan	eHealth Saskatchewan	No

^a^Available only upon request.

^b^BC: British Columbia.

^c^PEI: Prince Edward Island.

### Data Labeling

Data labeling (or tagging) is the common process of assigning one or more descriptive tags or labels to a dataset [32], which can make it easier to search and filter results while enabling other uses of the data (eg, machine learning) [33]. To provide an example of searchability in our proof-of-concept MDR, we identified potential language-related variables. We used a naive string-searching algorithm, which works by checking for the occurrence of a pattern (or string) at every possible position in the text [34]. Given Canada’s status as a bilingual English-speaking and French-speaking country, we identified potentially linguistic variables as those matching any of the following text strings: “french,” “english,” “lang,” “spoken,” “speak,” “ling,” and “franc.”

**Figure 1:**
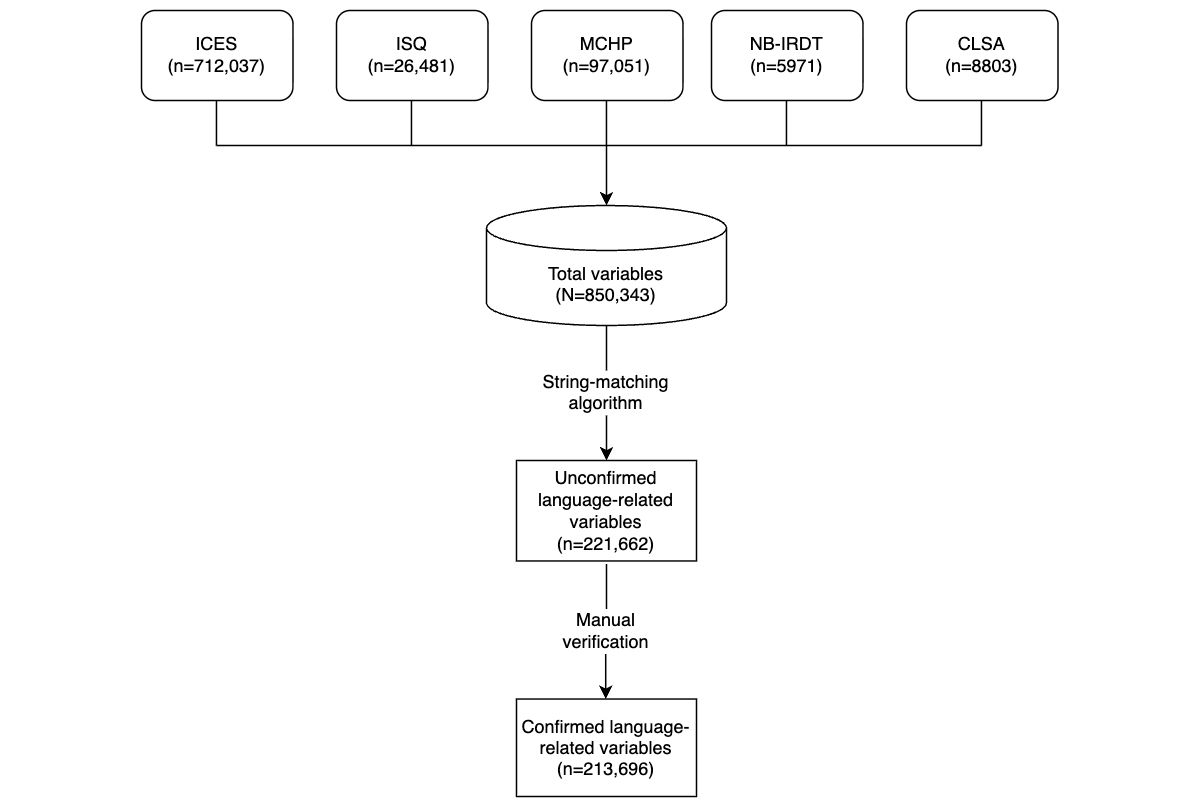
Flowchart for identification of language-related variables from health care data holdings. CLSA: Canadian Longitudinal Study on Aging; ICES: Institute for Clinical Evaluative Sciences; ISQ: Institut de la statistique du Québec; MCHP: Manitoba Centre for Health Policy; NB-IRDT: New Brunswick Institute for Research, Data and Training.

From here, 2 members of the research team (VM-S and CP) independently reviewed 9.9% (84,068/850,343) of the overall variable names and definitions in the dataset (these were taken from the list of potential language-related variables; Figure 1) to agree on the criteria to define what a language variable is, placing higher value on the most common definitions. The 2 researchers then met to reach a consensus on the standardized definition for tagging language-related variables within the proof-of-concept MDR: “any variable that directly or indirectly provides information regarding the linguistic characteristics of an individual, a health professional, or an organization.” This definition aimed to be extremely broad to be able to accommodate any form of research method, including Bayesian statistical approaches.

Both researchers then independently screened all previously identified variables, including variable names and definitions, to identify all language-related variables according to our standardized definition. Screening results were then compared to ensure consensus in the labeled variables between the 2 researchers. Any conflicts in the identification of language-related variables or in the application of the standardized tagging definition were resolved via conversation with a third member of the research team (LMB).

To quantitatively assess the reliability of this screening process, we calculated interrater reliability using Cohen κ [35], which measures the consistency with which both researchers independently applied the standardized definition, accounting for agreement that would be expected by chance. This approach is a standard practice in systematic reviews and content analysis methodologies where operational definitions are developed through iterative refinement and consensus-building discussion [36]. To reduce potential bias, we calculated Cohen κ on 137,594 of the 221,662 (62.0%) variables screened after the standardized definition was established (Figure 1), excluding the 84,068 (37.9%) variables from the initial screening phase used for definition development.

### Ethical Considerations

All data used in this study were limited to publicly available metadata, which posed no privacy risk or potential for harm. No personal health information, patient data, or confidential research data were accessed. The collected metadata consisted solely of variable names, descriptions, dataset structures, and data availability information—content that data custodians have chosen to make publicly available to facilitate research discovery and data access applications. For these reasons, approval from a research ethics board was neither required nor sought.

## Results

### Overview

Across the 5 included data sources, metadata from a total of 850,343 variables were collected and included in our repository. The number of metadata variables collected from each data holding is presented in Table 2. Among the data holdings, the ICES (712,037/850,343, 83.7%) and MCHP (n=97,051, 11.4%) data holdings contained the most variables.

Among the initial 850,343 variables in our repository, 221,662 (26.1%) potential or unconfirmed language-related variables were identified by using a search string algorithm across variable labels, names, and definitions. Consensus screening of these variables using a derived, standardized definition of language or linguistic variables identified 213,696 (25.1%) confirmed language-related variables in our repository (Figure 1).

Interrater reliability for the independent screening process was assessed using observed percent agreement and Cohen κ. The 2 researchers initially agreed on 96.3% (132,538/137,594) of these postdefinition variables, with 5056 (3.7%) disagreements that were subsequently resolved through consensus-building discussion. The calculated Cohen κ was 0.621 (95% CI 0.611-0.632), indicating substantial agreement between the 2 researchers.

**Table 2:** Number of metadata variables included in the proof-of-concept metadata repository by data holding (N=850,343).

Data holding	Variables, n (%)
Institute for Clinical Evaluative Sciences	712,037 (83.7)
Institut de la statistique du Québec	26,481 (3.1)
Manitoba Centre for Health Policy	97,051 (11.4)
New Brunswick Institute for Research, Data and Training	5971 (0.7)
Canadian Longitudinal Study on Aging	8803 (1.0)

### Creating a Usable Proof-of-Concept Web Interface

To facilitate exploration and use of the MDR, we developed a prototype web application that allows users to browse and search the MDR over the internet [37]. Although data management frameworks exist, such as the DataHub Project [38] and the Comprehensive Knowledge Archive Network (CKAN) [39], we developed our application in R (version 4.3.1; R Foundation for Statistical Computing) using *Shiny* [40]. *Shiny* is an open-source R package that makes it easy to build interactive web applications directly using R, a programming language widely used for statistical computing and graphics. *Shiny* was chosen due to its relative simplicity compared to other web development frameworks and the research team’s familiarity with the R programming language. The user interface of the *Shiny* app was designed with user-friendliness and functionality in mind, and it allows users to search by keyword, filter by data properties (eg, data holding and linguistic properties), and browse through paginated results. The *Shiny* application was built into a Docker image and hosted on a public platform-as-a-service provider.

## Discussion

### Principal Findings

Canada’s health data are scattered across many organizations and jurisdictions, each with its own policies and procedures, making it difficult for researchers to identify, locate, and use existing data [23,41]. To address this gap, we developed a proof-of-concept MDR containing metadata for more than 850,000 variables from 5 different Canadian data holdings and performed bottom-up labeling of 213,696 (25.1%) of the 850,343 language-related variables within the repository to help researchers easily identify language-related data within the vast landscape of Canadian health data. We also developed an openly accessible web application to allow users to search for the MDR [37].

### Building a Bottom-Up MDR: Lessons Learned

Our pilot project demonstrated the feasibility of a bottom-up approach to building an MDR for Canadian health data, but we learned several important lessons that we summarize here. First, complex, manual effort was required to collect (or “scrape”) data that are publicly available on the internet. Web scraping is very fast when it works, but each data source needs a bespoke approach. Some websites are straightforward to scrape (eg, those that use backend application programming interfaces) that can be queried directly), but others use an architecture that is not well suited to automatic data collection (eg, those that require repeated form submissions or client-side JavaScript). In addition, the scraping logic is custom-built to each website’s design at that moment in time, and if repositories update their websites, the scraping code will need to be updated as well.

Second, there is a need for robust internal data management practices when developing an MDR. We initially prioritized simplicity as well as data portability and transparency; therefore, we stored our data in plaintext CSV files. However, as we collected more data, we were surprised by the size of our final dataset, at a little more than 1 GB. Although this is small compared to many geospatial or genetic datasets, files of this size are unwieldy to work with, since common office software, such as Microsoft Excel, may not be able to load all variables and can be slow and difficult to transfer to others. For any similar projects, we suggest that a simple data-storage format, such as CSV files, is appropriate for initial feasibility studies, but the project should move quickly to a more sophisticated centralized data-storage solution (eg, a database or a large-file storage solution with version tracking) once feasibility has been established.

Finally, we learned that *Shiny* has several limitations that make it ill-suited for public-facing web applications with datasets this large. *Shiny* creates a new R session for each user and loads the entire dataset into server memory. For a typical dataset measured in KBs or MBs, the overhead is negligible; however, since our data are approximately 1 GB, our application runs out of memory and crashes with more than a few concurrent users. So, although *Shiny* was indispensable to us for rapid prototyping on a local computer, for production deployments, we suggest a different framework in which the data are stored in a single database and queried as needed, as opposed to the server loading a new in-memory copy of the dataset for each user. The user interface could be written using any web development framework (eg, Phoenix and React) and the open-source database software such as PostgreSQL, which is commonly used in large commercial and government projects, would be capable of handling queries on a million-row dataset with millisecond-level response times [42]. Direct access to the application programming interface could also be added, but implementation details of this potential future project are outside the scope of this paper.

### Limitations

Although our proof of concept provides a working example of a bottom-up labeled MDR, our methodology is not without limitations. For our initial screening of language variables, we used a search string algorithm to first identify potential language-related variables within all datasets in the proof-of-concept MDR. This search string may not have been exhaustive and could have missed potential language-related variables within the included datasets. Moreover, to best assess the accuracy of our algorithm, it would need to be tested against manually screened datasets as a gold standard for our definition. Although this process was considered too time consuming for the scope of this proof-of-concept project, given the size of the datasets used, it would allow us to evaluate measures such as sensitivity and specificity.

Finally, because variables in our repository were web scraped from various data sources, our repository reflects what variables were available at the point in time of data scraping and would require a repeat of the scraping, screening, and labeling process to update the repository as it is. In addition, there may have been additional metadata in the data holdings that were not made publicly available and therefore were not scrapable, meaning our proof-of-concept MDR may not be exhaustive of variables from the included data holdings. Nonetheless, without top-down policies and procedures in place to allow for easy data collection and labeling processes across Canada, our language-variable data labeling provides a working example of how bottom-up data labeling can be performed by researchers.

### Future Directions

Although currently in a beta version, we have plans to expand the MDR to include variables from additional Canadian administrative health data holdings, such as Population Data British Columbia [43], and data from Statistics Canada [44]. Moreover, additional variable tagging can be performed to identify sociodemographic variable types within all included datasets for research purposes, such as sex, gender, race, ethnicity, income, and immigration status. Regarding language-related variables specifically, subtagging can be performed for more specific variable definitions [45], including knowledge of official languages (French or English), variables indicating first language or mother tongue, or variables related to patient–health care provider language concordance.

We also intend to develop a new MDR web application that overcomes the limitations of our *Shiny* app by using a backend database, so that the entire dataset does not need to be loaded into server memory repeatedly for each user.

Finally, we believe that creating a *top-down* national MDR is a worthy goal that should be pursued in tandem with *bottom-up* efforts such as ours. However, such a project would face a number of governance, legal, ethical, and administrative barriers and require a high degree of alignment across diverse organizations so as not to create numerous delays in the collection and integration of data from provincial and organizational data custodians [46,47]. In other words, an ideal top-down MDR will need intense collaboration between many organizations, and although this is beyond our power as individual researchers, we hope Canada’s data custodians will rise to the challenge.

### Conclusions

This paper addresses the need for a national MDR of administrative and other health data in Canada, underscoring how an MDR can address issues caused by data fragmentation and increase the FAIRness of health care data across the country. However, complex challenges hinder the development of a top-down health data MDR in Canada. We developed a proof-of-concept MDR of administrative health data from 5 different data sources and performed bottom-up labeling of language-related variables within the repository to help researchers easily identify language data in the vast landscape of Canadian health data. This MDR is publicly available online as a searchable data dictionary [37].

Our proof-of-concept MDR illustrates the methodological limitations of a bottom-up approach, which can be complementary and synergistic with but cannot replace top-down approaches to the development of such a repository. Engagement of and collaboration between provincial data holdings and federal data agencies are critical to ensuring a pan-Canadian MDR is comprehensive and can be kept up to date. A national MDR would make it simple and straightforward for Canadian researchers to leverage the full scope of Canadian health data, and open opportunities for new studies as researchers discover datasets previously unknown to them. We believe that this could be transformative, and we hope this pilot project demonstrates the feasibility of a bottom-up approach in contributing toward this overarching goal.

## Appendix

Multimedia Appendix 1 Data collection methods and compliance by source.

## Abbreviations

CDP: Canadian Data Platform
CKAN: Comprehensive Knowledge Archive Network
DASH: Data Access Support Hub
FAIR: findability, accessibility, interoperability, and reusability
HDRN: Health Data Research Network
ICES: Institute for Clinical Evaluative Sciences
MCHP: Manitoba Centre for Health Policy
MDR: metadata repository
SPOR: Strategy for Patient-Oriented Research

## Data Availability

The data for this study are available for browsing [37], and the full dataset is available from the corresponding author on reasonable request.
