# Supplementary material for: Health Data for Linguistic Minority Group Research in Canada: Proof-of-Concept Centralized Health Care Metadata Repository Development and Usability Study
Source: JMIR Infodemiology. 2026 Feb 9;6:e77242. doi: 10.2196/77242 (PMC12930145; doi:10.2196/77242)
Supplement: Multimedia Appendix 1 [file infodemiology_v6i1e77242_app1.docx]

**Appendix 1. Data Collection Methods and Compliance by Source**

Metadata for this study were collected from five Canadian health data sources between January and September 2023. Our data collection employed three distinct methodological approaches, tailored to each source’s data accessibility, permissions structure, and technical infrastructure: (1) direct provision from data custodians, (2) programmatic downloading of publicly available CSV files, and (3) web scraping of public metadata pages. All data collection adhered to ethical web access practices, including verification of website access policies, appropriate rate limiting, and obtaining necessary permissions.

**1) Data Collection Methods**

Direct Data Provision (2 sources)

**Manitoba Centre for Health Policy (MCHP)** and **New Brunswick Institute for Research, Data and Training (NB-IRDT)** provided metadata files directly to our research team upon email request. No web scraping or automated data collection was performed for these sources. Data custodians at both institutions shared their metadata holdings as structured files (CSV format), eliminating any concerns regarding robots.txt compliance, rate limiting, or terms of service adherence. This direct data sharing represents the gold standard for metadata access and enabled the complete capture of their available metadata.

Programmatic CSV Download (1 source)

**Institut de la statistique du Québec (ISQ)** provides direct download links to CSV files for each dataset on their public data catalogue. We automated the retrieval of these publicly available files by scripting downloads from the URLs listed on their website. Example download URL:

https://statistique.quebec.ca/hmf-v2/hmf424_genr_fichr.p_genr_csv?p_lang=en&p_id_fichr=47

This approach accesses the same files available to interactive users clicking download buttons on each dataset page, rather than parsing HTML content. The URL structure includes parameters for language selection (p_lang=en) and file identification (p_id_fichr=47).

**Compliance verification:**

We reviewed ISQ’s robots.txt file, which was captured in December 2023:

# robots.txt from https://statistique.quebec.ca/robots.txt
User-agent: *
Disallow: /pls/ken/
Disallow: /statistiques/recensement/
Allow: /
Sitemap: https://statistique.quebec.ca/sitemap.xml

**Interpretation:** Our download path (/hmf-v2/) does not match either restricted directory (/pls/ken/ for database backend or /statistiques/recensement/ for census data) and falls under the explicit “Allow: /” directive. This method represents programmatic access to publicly available files through their intended download mechanism.

Web Scraping from Public Sources (2 sources)

For **Institute for Clinical Evaluative Sciences (ICES)** and **Canadian Longitudinal Study on Aging (CLSA)**, we performed web scraping of publicly accessible metadata. All scraping was conducted in accordance with established ethical web data collection practices.

***ICES (Ontario)***

**Source:** ICES Data Dictionary (https://datadictionary.ices.on.ca/)

**Method:** Web scraping performed using R programming language to extract metadata from publicly accessible data dictionary pages.

**Compliance verification:**

- **robots.txt status:** No robots.txt file exists at https://datadictionary.ices.on.ca/robots.txt (HTTP 404 error returned). Per web standards, the absence of a robots.txt file indicates no restrictions on automated access.
- **Public access design:** The ICES Data Dictionary explicitly states it is “easily accessed from inside and outside of ICES” and distinguishes between publicly available metadata (accessible to all users) and internal technical details (restricted to ICES personnel). We scraped only the publicly accessible metadata visible to external users.
- **Terms of service compliance:** ICES Terms of Use (last modified September 2023) permit use of publicly accessible content subject to fair dealing principles. For non-commercial research purposes such as this study, ICES requests notification 30 days before publication. We obtained verbal permission from ICES administrators prior to data collection and publication.
- **Technical safeguards:** No anti-bot measures (e.g., CAPTCHA, rate limiting, IP blocking) were present on the public data dictionary, and no authentication was required to access the metadata.

**Permission:** Verbal permission obtained from ICES administrators in 2023, prior to data collection.

***CLSA (National)***

**Source:** CLSA website (https://www.clsa-elcv.ca/)

**Method:** Web scraping performed using R programming language to extract publicly available metadata about study variables.

**Compliance verification:**

The CLSA website has a fully permissive robots.txt file, captured in December 2023:

# robots.txt from https://www.clsa-elcv.ca/robots.txt
# START YOAST BLOCK
# ---------------------------
User-agent: *
Disallow:

Sitemap: https://www.clsa-elcv.ca/sitemap_index.xml
# ---------------------------
# END YOAST BLOCK

**Interpretation:** An empty Disallow: directive (with no paths specified) indicates that all content is explicitly permitted for automated access by any web crawler. This represents a fully permissive stance on web scraping of public content.

- **Public access:** All metadata scraped was publicly accessible without authentication requirements.
- **Technical safeguards:** No anti-bot measures were present on the public metadata pages.

**2) Technical and Ethical Practices**

Understanding robots.txt Compliance

The robots.txt file is a standard protocol (Robots Exclusion Protocol) used by websites to communicate access policies to automated web crawlers. A Disallow: directive specifies URL paths that should not be accessed by automated tools; an empty Disallow: means no restrictions apply. The absence of a robots.txt file (HTTP 404 error) indicates the website has not imposed any access restrictions on automated access.

**Note:** robots.txt files displayed in this appendix represent website access policies at the time of data collection (2023). Future researchers should verify current policies before reproducing this methodology, as website access rules may change over time.

Rate Limiting

All web scraping operations were performed using R’s default HTTP request behavior, which includes:

- Sequential requests with natural delays between page loads
- No parallel or concurrent requests that could overwhelm servers
- Respectful request patterns that mimic human browsing behavior
- Adherence to standard HTTP protocol timeouts and retry logic

No aggressive or high-frequency scraping techniques were employed. Total data collection occurred over several weeks (January–September 2023) to minimize server load impact.

Authentication and Access Restrictions

We did not attempt to:

- Bypass authentication systems or login requirements
- Circumvent paywalls or subscription barriers
- Access password-protected or restricted content
- Access content marked as private, internal-only, or confidential
- Use automated tools to repeatedly access content after receiving error responses

All data collection was limited to publicly accessible metadata that data custodians had chosen to make available without authentication.

Data Collection Timeframe

Metadata reflects the state of each data source at the time of collection (January–September 2023). As data holdings continuously evolve (new datasets added, variables updated, documentation revised), our repository represents a point-in-time snapshot rather than a continuously updated resource. Future updates would require re-collection using the same methods described above.

Reproducibility Considerations

Researchers wishing to reproduce or extend our work should note:

1. **Direct provision sources (MCHP, NB-IRDT):** Contact data custodians directly via email to request current metadata files. Contact information is available on their institutional websites.
2. **Programmatic CSV download (ISQ):** Direct download links remain publicly accessible on the ISQ website as of this publication. Updated metadata can be retrieved by collecting current download URLs from dataset pages and scripting the downloads.
3. **Web scraping sources (ICES, CLSA):**

- Verify current robots.txt compliance before initiating any web scraping
- Review and respect any updated terms of service or access policies
- Consider requesting direct data provision or API access if available, as this reduces technical complexity
- Website structures and HTML layouts may have changed since 2023, requiring updated scraping code
- Seek permission from data custodians when in doubt about appropriate access methods

1. **Technical tools:** All data collection used R programming language (version 4.x) with standard HTTP libraries (mainly httr, rvest packages). Specific scraping code is available from the authors upon request.

Ethical Statement

All data collection was limited to publicly available metadata that poses no privacy risk or potential for harm. No personal health information, patient data, or confidential research data were accessed. The metadata collected consists solely of variable names, descriptions, dataset structures, and data availability information—content that data custodians have chosen to make publicly available to facilitate research discovery and data access applications.

Data Provenance and Attribution

All metadata remains the intellectual property of the respective data custodians. This repository does not claim ownership of the metadata content but serves as an aggregated catalogue to improve the discoverability of Canadian health data resources. Users of this metadata repository should:

- Cite the original data custodians when referencing specific datasets or variables
- Respect all use restrictions and access procedures established by data custodians
- Contact data custodians directly for access to the underlying health data (this repository contains only descriptive metadata, not the health data itself)

Contact Information for Data Custodians

For access to current metadata or underlying health data:

- **ICES (Ontario):** https://www.ices.on.ca/
- **MCHP (Manitoba):** https://umanitoba.ca/manitoba-centre-for-health-policy/
- **ISQ (Québec):** <https://statistique.quebec.ca/en>
- **NB-IRDT (New Brunswick):** https://www.unb.ca/nbirdt/
- **CLSA (National):** https://www.clsa-elcv.ca/
